# Fluorescence molecular imaging of high-grade gliomas and brain metastases using the RAS70 peptide targeting plasma membrane-bound Hsp70 on tumor cells

**DOI:** 10.1007/s11060-025-05245-0

**Published:** 2025-10-15

**Authors:** Anastasiia Nechaeva, Alexei Ulitin, Daria Sitovskaya, Natalia Yudintceva, Danila Bobkov, Ruslana Likhomanova, Victor Olyushin, Aleksander Kim, Konstantin Samochernykh, Stephanie E. Combs, Maxim Shevtsov

**Affiliations:** 1 Polenov Neurosurgical Institute, Almazov National Medical Research Centre, Mayakovskogo Str. 12, St. Petersburg, 191014 Russia; 2 Personalized Medicine Centre, Almazov National Medical Research Centre, Akkuratova Str. 2, St. Petersburg, 197341 Russia; 3 Laboratory of Biomedical Nanotechnologies, Institute of Cytology of the Russian Academy of Sciences (RAS), Tikhoretsky Ave. 4, St. Petersburg, 194064 Russia; 4 Department of Radiation Oncology, Klinikum rechts der Isar, Technical University of Munich, Ismaninger Str. 22, 81675 Munich, Germany

**Keywords:** Membrane-bound Hsp70, Glioblastoma, Brain metastases, Fluorescence-guided surgery, Targeted diagnostics

## Abstract

**Background:**

One of the promising targets for fluorescence-guided surgery of malignant brain tumors is a 70 kDa heat shock protein (mHsp70), which has been found on the plasma membrane of tumor cells. In this study, we conjugated the RAS70 peptide, which targets mHsp70 on tumor cells, with the fluorophore Cy7.5 and used it for epifluorescence detection of mHsp70-positive glial tumors and brain metastases.

**Methods:**

The study included adult patients with newly diagnosed glioblastoma (GBL, *n* = 7) and brain metastases (MTS, *n* = 8). Three hours prior to surgery, patients received an oral solution of 5-aminolevulinic acid (5-ALA, 20 mg/kg). During surgery, tumor samples were obtained from three zones: necrotic, viable tumor, and perifocal. Some samples were treated ex vivo with the RAS70-Cy7.5 peptide, while others were treated with the control peptide NGL-RGD-Cy7.5. Epifluorescence images were obtained using an operating microscope in FL400 and FL800 modes, image analysis was performed in ImageJ with calculation of the target-to-background ratio (TBR). The presence of mHsp70 on the tumor cell membrane was additionally determined using immunofluorescence microscopy.

**Results:**

Immunofluorescence staining revealed mHsp70-positive tumor cells in all studied samples, with preferential localization observed in the viable (contrast-enhancing) and perifocal tumor zones. TBRs for fluorescence imaging of RAS70-Cy7.5 peptide were significantly higher than those for the control peptide (*p* < 0.0001): necrotic zone 19.2 a.u. (15.5–21.0), viable tumor 9.0 a.u. (7.0–11.5), perifocal zone 8.9 a.u. (6.7–11.4), control – 1.2 a.u. (0.9–1.7). RAS70 exhibited 37.5% more visible fluorescence than 5-ALA in viable metastasis zones (*p* < 0.001). The RAS70 peptide was more sensitive and specific than 5-ALA for detecting perifocal zones in glioblastomas and brain metastases (100% vs. < 75% and 100% vs. < 85%, respectively).

**Conclusion:**

The RAS70 peptide demonstrated high diagnostic accuracy for glioblastoma and metastases, suggesting potential applications in intraoperative image-guided tumor resection and the development of targeted drug delivery systems.

## Introduction

Malignant glial tumors and brain metastases of solid tumors are characterized by poor prognosis for the life of patients due to the high risk of local and distant relapse, despite complex treatment approach [1, 2]. It has been proven that performing the most radical tumor resection is associated with a more favorable prognosis for the disease [3–6]. However, this is not always possible because the tumor cells enter adjacent healthy brain tissue, making it difficult to determine the tumor boundaries, and microscopic residual tumor cells are preserved even after complete resection.

One of the promising technologies that allows us to determine tumor tissue from normal brain is intraoperative fluorescence imaging. The principle of fluorescence visualization is based on the use of fluorescence agents that are fixed in tumor cells using various mechanisms of action [7]. Currently, there are a limited number of drugs approved for clinical use in neuro-oncology. First of all is 5-aminolevulinic acid (5-ALA), a precursor in the heme biosynthesis pathway that is metabolized to protoporphyrin IX. This agent has undergone three phases of clinical trials and was approved by the FDA in 2017 [8, 9]. The 5-ALA as a fluorescence agent with a metabolic type of action has certain disadvantages. For cerebral metastases, the rates of visible 5-ALA fluorescence range from 27.6 to 86.9% and depended on the histologic tumor type [10]. This fact limits the use of 5-ALA in the resection of metastases without the use of additional equipment that allows for a quantitative assessment of 5-ALA accumulation. Furthermore5-ALA has a wide range of sensitivity (70 to 79%) and specificity (75 to 100%) in differentiating glioblastoma tissue from healthy brain tissue [11–13]. One of the reasons for such results is the influence of the autofluorescence effect of the background (normal brain tissue), which contains normal tissue chromophores (e.g. collage, flavins), which have a similar spectral characteristics as protoporphyrin IX (excitation: 400 nm, emission: 635 nm) that accumulates in tumor cells after the administration of 5-ALA [14].

Improving the accuracy of intraoperative diagnostics of residual malignant brain tumors can be achieved by developing molecular imaging methods, which is one of the most important areas of translational and clinical oncology. The choice of tumor-specific target is a key factor in the effectiveness of such drugs. The selected target must be present in the majority of the tumor cell population and be accessible to the target drug. Heat shock protein 70 kDa (Hsp70) is such a tumor-specific target, as it is expressed on the surface of the plasma membrane of various malignant tumors [15–17], including glioblastoma cells [18, 19]. The tumor-specific localization of membrane-bound Hsp70 (mHsp70) could be explained by the association of the chaperone with certain lipids in the lipid rafts of the plasma membrane (i.e., globotriaosylceramide Gb3) or with negatively charged phosphatidylserine [20, 21].

In the previous studies employing live-cell immunofluorescence microscopy of high-grade glioma tissues (*n* = 32) and brain metastases (*n* = 3) it was demonstrated the presence of mHsp70-positive cells both in the contrast-accumulating zones of the tumor and in the perifocal zone [22]. In contrast, studies of brain tissue obtained from patients with drug-resistant epilepsy did not reveal the presence of mHsp70-positive cells [22]. The high presence of the membrane-bound chaperone was associated with an increased migration and invasion of the tumor cells which could be efficiently suppressed by Hsp70-targeted inhibitors (e.g., PES, JG-98), which in turn resulted in the favorable overall survival of glioma-bearing animals [22, 23].

In the current study, for tumor molecular imaging we employed infrared Cy7.5-labeled mHsp70-targeted peptide TKDNNLLGRFELSG-Beta-Ala-RGD termed RAS70, which is known to bind to the oligomerization sequence of mHsp70 chaperone [24]. Previous preclinical studies on various glioma cell lines have shown that the peptide recognizing mHsp70 has demonstrated high tumor-homing properties when being either intravenously administered or locally applied in the orthotopic glioblastoma models [24]. Thus, being locally sprayed over the dissected brain tissues RAS70-sCy7.5 peptide helped to efficiently delineate the tumors in glioma-bearing animals employing an intraoperative fluorescence imaging system, which was further confirmed by histological studies [24].

In this single-center, open-label, non-blinded prospective study, we investigated the diagnostic accuracy and translational applicability of the fluorescently labeled peptide RAS70-Cy7.5 for fluorescence imaging of malignant gliomas and brain metastases during surgical resection. We compared its fluorescence imaging to that of 5-ALA and a scrambled peptide (NGL-RGD-Cy7.5). Our results indicate that this mHsp70-targeted peptide may be useful for the effective delineation of tumors from adjacent normal brain tissue, particularly in the perifocal zone.

## Methods

### Participants

The study was conducted on biopsy material obtained during surgical treatment of adult patients with suspected (according to preoperative MRI diagnosis) newly diagnosed high-grade gliomas (*n* = 7) and brain metastases (*n* = 8) without previous radiation therapy and chemotherapy, with a Karnofsky performance scale of 60 and more, with no renal insufficiency (i.e. creatinine < 177 µmol/L) and hepatic insufficiency (i.e. gamma-glutamyl transpeptidase < 100 U/L). Patients with tumor localization in functionally significant areas of the brain, which limits the availability of sufficient samples for examination, were excluded from the study. All patients received freshly prepared solutions of 5-aminolevulinic acid (Alasens, State Scientific Center NIOPIK, Moscow, Russia) orally (20 mg/kg) 3 h (range 2–4) prior to surgery. To evaluate the tumor-specific properties of mHsp70, intraoperative samples from patients with drug-resistant epilepsy due to focal cortical dysplasia (*n* = 2) were utilized.

Study protocol was approved by the Ethics Committee of the Almazov Medical Research Centre (approval No. 11–24 from 25.11.2024) and was conducted in accordance with the Declaration of Helsinki. Written informed consent was obtained from all participants. All studies were conducted in compliance with all applicable guidelines and regulations.

### Radiologic evaluation

**Fig. 1:**
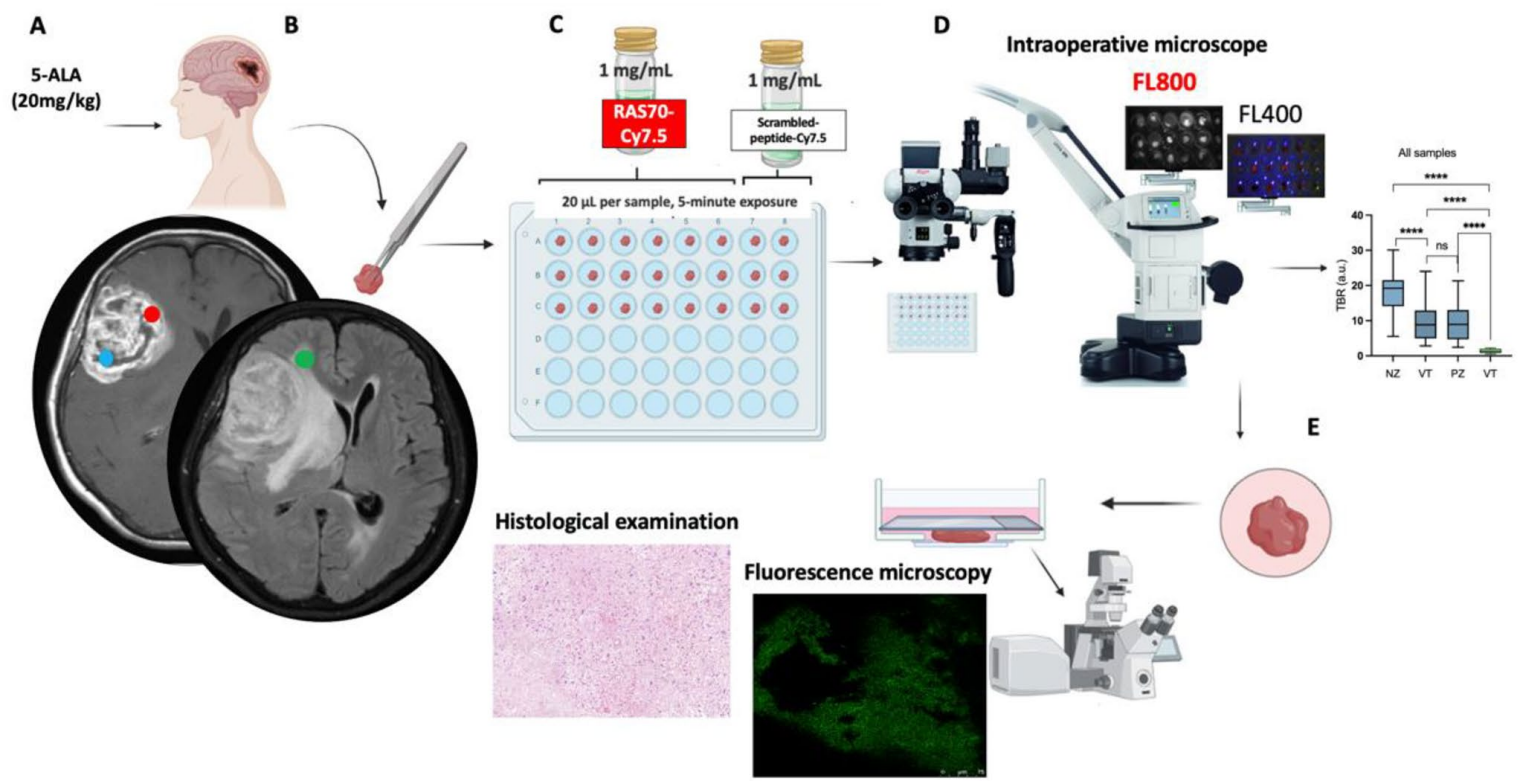
Study design. (**A**) Taking 5-aminolevulinic acid (5-ALA, 20 mg\kg). (**B**) Obtaining tumor samples from three tumor zones: necrotic (blue circle), viable tumor (red circle), perifocal (green circle). (**C**). Spraying of samples with RAS70-Cy7.5 and NGL-RGD-Cy7.5 peptides (**D**) Ex vivo fluorescence imaging using the intraoperative microscopes (Leica M720 and M530, Wetzlar, Germany) and fluorescence quantification with ImageJ Washington, DC, USA. (**E**) Histological examination and fluorescence microscopy samples

Before surgery, all patients underwent contrast-enhanced MRI of the brain on an expert-class tomograph (MAGNETOM Skyra 3.0T, Siemens, Munich, Germany). Scanning and image analysis were performed in accordance with the standard imaging protocol [25]. Scanning was performed in the axial plane in T1, T2 FLAIR, DWI modes (slice thickness 1 mm), as well as in the axial sagittal and coronal planes in T1 mode with contrast enhancement (slice thickness 1 mm). Based on the performed MRI, areas for subsequent biopsy collection for ex vivo study were determined, as previously proposed by Hubert et al. 2016 [26]. These regions were: necrotic zone - hypoxic core, hypointense on T1 and non-enhancing tumor area (the blue circle on Fig. 1B); viable tumor zone - the contrast enhanced tumor zone (the red circle on Fig. 1B); perifocal zone - the potentially invasive FLAIR region, ≥ 3 mm from enhancing margin (the green circle on Fig. 1B).

### Synthesis and conjugation of the RAS70 targeted peptide with fluorochromes

The RAS70 peptide (TKDNNLLGRFELSG-Beta-Ala-RGD) was obtained by solid-phase peptide synthesis using Fmoc technology on Wong’s polymer. The NGL-RGD scrambled peptide (NGLTLKNDFSRLEG Beta-Ala-RGD), obtained by a similar method, was selected as a control peptide. Fmoc protection was removed with a 20% piperidine solution in dimethylformamide, and 1,3-diisopropylcarbodiimide with the addition of hydroxybenzotriazole was used as a condensing agent. Removal from the resin was performed with trifluoroacetic acid (TFA) with the addition of scavengers. The peptide was purified by RP-HPLS in an acetonitrile gradient in 0.1% TFA in water on a chromatographic column Xbridge BEH130 prep C18 OBD 10 mm 130 A 250*19 mm (Waters, Milford, MA, USA) to a purity of at least 95%, followed by lyophilization. Purity analysis was performed by RP-HPLS in a gradient of acetonitrile in 0.1% TFA in water on a Waters Delta-Pak 5 mm 100 A 150*3.9 mm analytical chromatographic column (Waters, Milford, MA, USA). The authenticity of the obtained peptide was confirmed by mass spectrometric analysis.

After purification, the RAS70 peptide was conjugated to fluorophores: sulfo-Cy7,5-NHS (CAS 2736437-44-6, Cat. No. 66320, excitation / emission: 800 nm / 820–900 nm, Lumiprobe, Westminster, Maryland USA) for intraoperative fluorescence microscopy and Alexa Fluor 488 (cat. No. A10235, excitation/emission: 488 nm / 496 nm, Thermo Fisher Scientific, Waltham, MA USA) for immunofluorescence microscopy. The control scrambled peptide NGL-RGD was conjugated to the fluorophore sulfo-Cy7,5-NHS (CAS 2736437-44-6, Cat. No. 66320, excitation/emission: 800 nm / 820–900 nm, Lumiprobe, Westminster, Maryland USA). Conjugation was performed in 0.1 M sodium carbonate buffer pH 8.5 with 10% DMSO for 3 h in the dark, the peptides were taken in a 4-fold molar excess with respect to the fluorophore. Purification and characterization of the conjugates were carried out using the same methods and the same equipment as for the free peptide. The purified conjugate was lyophilized and stored at − 20 °C, reconstituting immediately before use.

### Intraoperative ex vivo imaging and fluorescence quantification

On the day of surgery biopsies were taken from three tumor zones identified according to preoperative MRI of the brain: necrotic, viable, and perifocal zones (3–4 samples from each zone, sample volume 30–50 mm³) (Fig. 1B). All samples were obtained without the use of bipolar coagulation. Each sample was placed in a separate microplate well and labeled (Fig. 1C). In the operating room immediately following samples taking we assessed the fluorescence of protoporphyrin IX (5-ALA) using intraoperative microscopes (Leica M720 and M530, Wetzlar, Germany) in FL400 mode. Then the part of samples was sprayed with peptide RAS70-Cy7.5 conjugated (20 µl per sample) and the exposure time constituted 5 min at room temperature. Another part of samples from viable tumor zone was sprayed with control NGL-RGD-Cy7.5 conjugated peptide (20 µl per sample) and the exposure time constituted 5 min at room temperature. Following rinsing of the samples with PBS solution the fluorescence images were obtained in the operating room using the same intraoperative microscopes (Leica M720 and M530, Wetzlar, Germany) in FL800 mode (Fig. 1D).

The fluorescence signal displayed was recorded using a microscope camera. Surgical specimens were fixed with 4% paraformaldehyde (pH = 7.0) for subsequent histological examination and fluorescence microscopy. The RAS70-Cy7.5 fluorescence information was not used to guide resection. Fluorescence imaging contrast was analyzed with ImageJ software (version 1.54 g, Washington, DC, USA) in circular ROIs (diameter: 50 pixels) corresponding to histologically confirmed tumor zones with Target-to-Background ratio (TBR) determination. Target-to-Background ratio (TBR) was determined by dividing the mean fluorescence value of target regions of interest (ROIs) by the mean signal intensities of background ROIs.

### Histological examination

Histopathological tumor typing and grading were based on the standard criteria of the 5th edition of the World Health Organization (WHO) classification of central nervous system (CNS) tumors (2021) [27]. In the pathology department, the material was fixed with 10% buffered paraformaldehyde in 0.1 M sodium phosphate buffer (pH = 7.0). Material was dehydrated in a standard manner and embedded in paraffin. Histological sections stained with hematoxylin and eosin were studied, as well as the results of immunohistochemical (IHC) reactions with the following antibodies for glioma: anti-IDH1R132H (1:300, Dia-H09, Dianova, Hamburg, Germany), anti-ATRX (1:300, ab188029, Abcam, MA 02453, USA), anti-EGFR (1:100, ab52894, Abcam, MA 02453, USA), anti-MGMT (1:300, NB100-168, Novus Biologicals, Colorado, USA), anti-Ki67 (1:50, M7240, Dako, CA 93013, USA). For metastasis we used anti-pan-CK (1:50, M0821, Dako, CA 93013, USA), anti-p40 (RTU, PDR055, Diagnostic BioSystems, CA 94588, USA), anti-TTF1 (1:50, Mob285, Diagnostic BioSystems, CA 94588, USA), anti-ER (RTU, IR657, Dako, CA 93013, USA), anti-PR (RTU, RMPD002, Diagnostic BioSystems, CA 94588, USA), anti-PAX8 (RTU, RDM180, Diagnostic BioSystems, CA 94588, USA). The EnVision polymer detection system (Dako, CA 93013, USA) was also used. For visualization, the streptavidin-peroxidase polymer ultrasensitive system and DAB chromogen (Sigma-Aldrich, Darmstadt, Germany) were used. The sections were counterstained with Gill’s hematoxylin and then embedded in Bio Mount HM synthetic embedding medium (BIO-OPTICA, Milano, Italy). Additionally, reactions lacking primary antibodies were carried out to ensure the specificity of the observed staining. Histological analysis and microphotography were performed using a scanning microscope Leica Aperio AT2 and the image manager AperioImageScope (Leica Microsystems, IL 60089, Wetzlar, Germany). Quantification of the results of IHC reactions was performed by counting positively stained cells in sections of the brain tissue (х400/200 in 1 mm^2^ slices, ImageJ).

### Immunofluorescence microscopy

For Hsp70 detection, intraoperative tumor samples (30–50 mm³) as well as samples obtained from patients with focal cortical dysplasia were pre-visualized using intravital confocal microscopy. The material was washed with PBS and stained for 1 h with monoclonal Hsp70 antibody conjugated to FITC (SPA810, Stress-Marq Biosciences Inc, Victoria, Canada), TMRM (tetramethylrodamine methyl ester) dye (1 µmol/L, T668, Thermo Fisher Scientific, Waltham, Massachusetts, USA) to confirm tissue viability and Hoechst 33,342 (0.1 mg/mL, H1399, Invitrogen, Waltham, Massachusetts, USA) to visualize nuclei. The samples were then washed and placed in a thin-bottomed Ibidi µ-Dish 35-mm (80136, Ibidi, Gräfelfing, Germany) and covered with a cover glass. The material was visualized by Leica TCS SP8 (Leica Microsystems, Wetzlar, Germany) fitted with argon and helium-neon lasers. An HC PL APO 63x/1.40 OIL CS2 oil immersion lens was used. Images were obtained at a resolution of 1024 by 1024 pixels with an average of three along each scanning line. Throughout the experiment, the same system settings were used. Images of unstained samples were used as a control for autofluorescence. At least 10 images were obtained from each sample, and the average intensities of green (Hsp70) pixels were calculated from confocal images using ImageJ software (NIH, Washington, USA).

From tissue microfragments fixed in formalin for 24 h - necrotic, viable and perifocal zones (2–3 samples from each zone) - histological sections were prepared on a Leica CM 1510 S freezing microtome for subsequent immunofluorescence examination (Leica Microsystems, IL 60089, Wetzlar, Germany). The thickness of the sections was 5–7 μm, the sections were mounted on glasses with an adhesive coating (poly-L-lysine).

Fixed tissue sections were permeabilized with 0.1% Triton X-100 (Servicebio, Wuhan, China), blocked with 5% bovine serum albumin (A9418, Sigma-Aldrich, Burlington, Massachusetts, USA), and stained overnight with the peptide RAS70-Alexa Fluor 488 (75 µg/mL) and monoclonal Hsp70 antibody conjugated to Alexa Fluor 555 (1:100, EP1007Y, ab223389, Abcam, Cambridge, UK). After washing, tissue sections were mounted in Mounting Medium with 4′,6-diamidino-2-phenylindole (DAPI, 50011, Ibidi, Gräfelfing, Germany). The tissue was visualized by Leica TCS SP5 Apo confocal laser system (Leica Microsystems, Wetzlar, Germany) equipped with argon and helium-neon lasers at wavelengths of 405, 488, and 543 nm. An oil immersion lens, HC PL APO 40x/1.30 Oil CS2, was employed.

### Statistics

Statistical analysis was performed using the Prism GraphPad 10 program (GraphPad Software, USA). The normality test was performed using Kolmogorov-Smirnov, Shapiro-Wilk tests. Average values of quantitative features are presented as medians and 95% CI. Group comparisons of quantitative and nominative features were performed using the Mann-Whitney U-test and Fisher, respectively. The diagnostic value of the RAS-Cy7.5 was assessed by calculating the area under the curve (AUC) obtained from the receiver operating characteristic (ROC) curve. The differences were considered statistically significant at р<0,05. The resulting graphs were performed using the Prism GraphPad 10 program (GraphPad Software, Boston, MA, USA).

## Results

### Patient characteristics

Between November 2024 and May 2025, 15 patients were enrolled of the ex vivo prospective study. Patient characteristics are presented in Table 1. The average age of enrolled patients constituted 64 years (range 42–69) in high-grade gliomas cohort (HGG; *n* = 7) and 62 years (range 33–84) in brain metastasis cohort (MTS; *n* = 8). Most of the tumors were located in the frontal (30%) and occipital (30%) lobes of the brain. From each patient, 3–4 samples were collected from each of the three tumor zones. As a result, 138 samples of tumor tissue were obtained for exposing to the RAS70 peptide. Of these, 66 samples were from HGG (*n* = 27 VT, *n* = 20 PZ, *n* = 19 NZ) and 72 from MTS (*n* = 24 VT, *n* = 25 PZ, *n* = 23 NZ). Additionally, 40 samples from viable tumor zones (20 from each group of patients) were obtained for exposing to the control NGL-RGD-Cy7.5 peptide. Fluorescence of obtained tumor samples and perifocal zone were measured in the operating room before the specimens were submitted for pathology investigation. On final pathology diagnosis and grading performed using the 2021 WHO criteria, glioblastoma (isocitrate dehydrogenase IDH1 wildtype, WHO Grade 4) was confirmed in seven (100%) patients in high-grade gliomas cohort. Metastases of poorly differentiated breast carcinoma (37.5%), lung cancer (25%), poorly differentiated uterine carcinoma (12.5%), colon adenocarcinoma (12.5%), ovarian cancer (12.5%) have been diagnosed in the brain metastasis cohort.

**Table 1:** Patients and tumor characteristics

	Age (years)	Sex	Tumor site	Histopathological Diagnosis	Dex. before surgery (mg)
**High-grade gliomas cohort**					
Patient 1	42	male	right occipital	glioblastoma IDH-wt	12
Patient 2	66	female	left occipital	glioblastoma IDH-wt	8
Patient 3	69	female	right frontal	glioblastoma IDH-wt	12
Patient 4	68	female	left frontal	glioblastoma IDH-wt	8
Patient 5	58	male	left parietal	glioblastoma IDH-wt	8
Patient 6	64	female	right frontal	glioblastoma IDH-wt	8
Patient 7	53	female	right frontal	glioblastoma IDH-wt	8
**Brain metastases cohort**					
Patient 8	60	female	right parietal	breast carcinoma metastasis	4
Patient 9	73	female	right occipital	uterine carcinoma metastasis	8
Patient 10	84	female	right temporal	colon adenocarcinoma metastasis	12
Patient 11	59	female	left frontal	breast carcinoma metastasis	12
Patient 12	77	female	right cerebellar hemisphere	ovarian cancer metastasis	8
Patient 13	33	female	right cerebellar hemisphere	breast carcinoma metastasis	4
Patient 14	64	male	right occipital	squamous cell lung cancer metastasis	4
Patient 15	42	male	right occipital	lung adenocarcinoma metastasis	8

Dex. = Dexametazone, wt = wild type

### Expression of the mHsp70 in the glioblastomas and brain metastasis

**Fig. 2:**
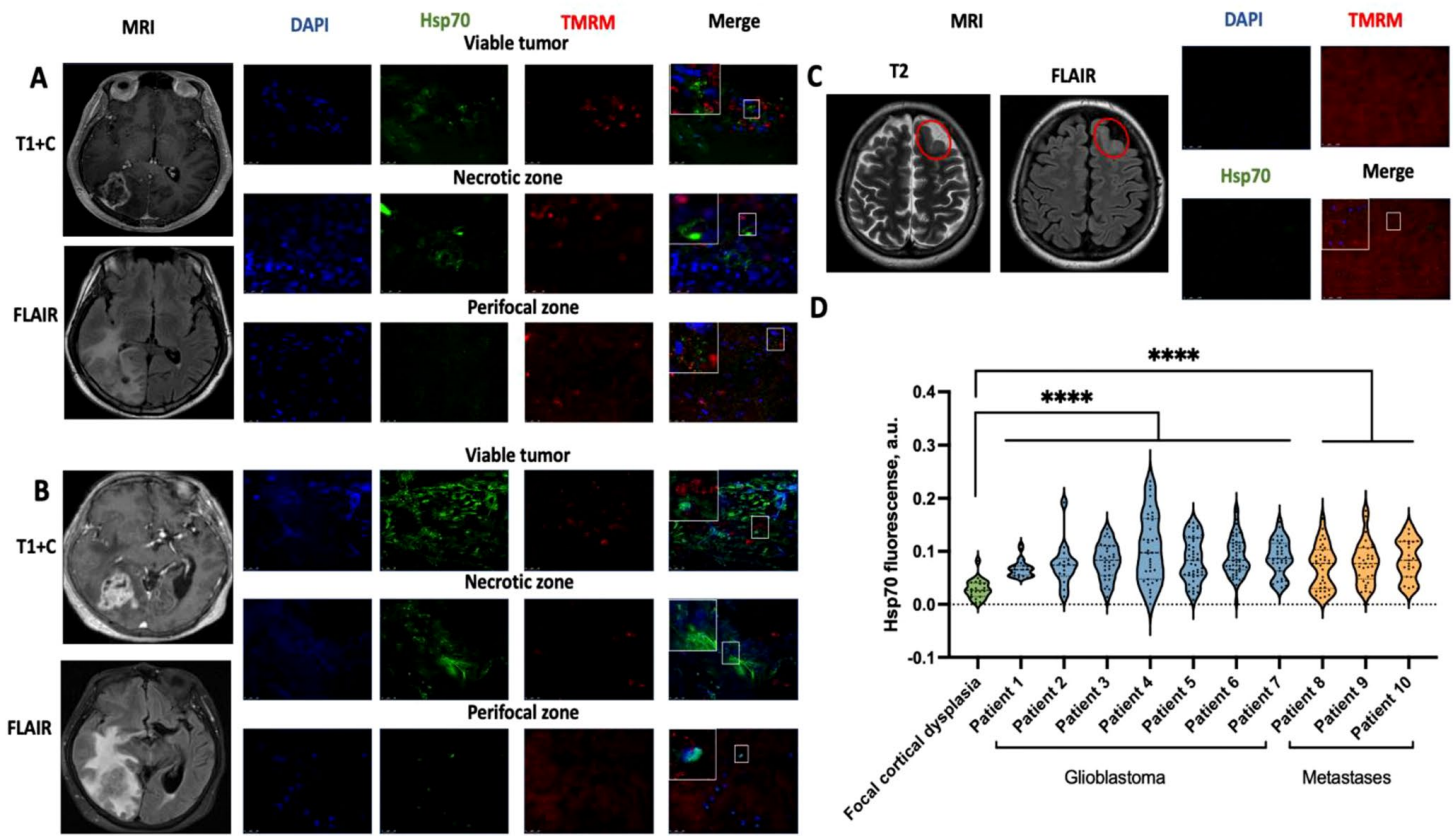
(**A**) Representative confocal microscopy images and respective MR images of the high-grade glioma (Patient #1 are presented). (**B**) Representative confocal microscopy images and respective MR images of the brain metastasis (Patient #9). (**C**) Representative confocal microscopy images and respective MR images of the focal cortical dysplasia. The area of focal cortical dysplasia is highlighted by a red circle. (**D**) Fluorescence intensity measurements of mHsp70 expression in live-cell microscopy images of viable zone of glioblastoma (*n* = 7) and brain metastasis (*n* = 3). The violine plot shows the median ± 95% CI (Man–Whitney test result **** *p* ≤ 0.0001). Hsp70 - green, TMRM (staining of cell mitochondria) – red, DAPI (staining of cell nuclei) – blue

Evaluation of mHsp70 expression in viable tumor cells from glioblastoma (*n* = 7) and brain metastasis (*n* = 3; Fig. 2) biopsies revealed a high degree of heterogeneity. Heterogeneous mHsp70 expression was observed in both viable and perifocal zones. Figure 2A shows representative images of biopsy material from a patient with glioblastoma in which mHsp70-positive tumor cells were detected. In addition, we examined chaperone expression in patients (*n* = 3) with brain metastases (Fig. 2B), in which mHsp70 expression was also detected in both the viable (contrast-enhanced) and perifocal (FLAIR region) tumor zones.

As a control, biopsy material obtained from patients operated on for drug-resistant epilepsy (*n* = 2; Fig. 2, C) was examined. Microscopic examination of viable cells revealed weak intracellular expression of Hsp70, with no evidence of membrane localization.

### Intraoperative fluorescence ex vivo imaging using peptide RAS70-Cy7.5, control scramble peptide NGL-RGD-Cy7.5 and 5-ALA on glioblastomas samples

**Fig. 3:**
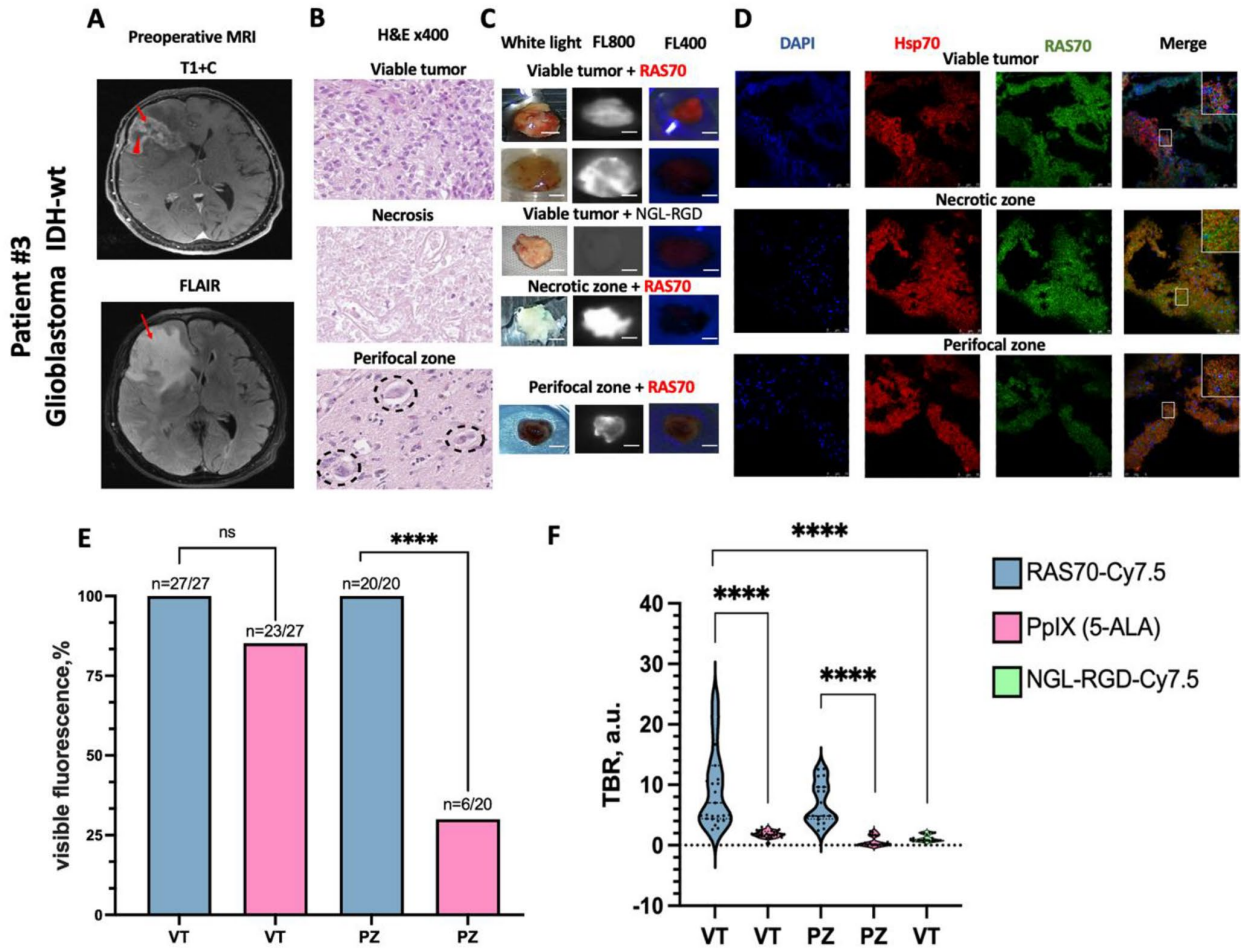
Representative intraoperative epifluorescence ex vivo imaging of high-grade glioma (Patient #3) (**A**) Representative preoperative MR images show the sampling sites: on T1 + C image, the red arrow indicates the area of viable tumor tissue sampling, the red arrowhead indicates the area of necrosis, on the FLAIR image, the red arrowhead indicates the perifocal zone. (**B**) Representative histological images of viable tumor, necrosis, perifocal zone. H&E = Hematoxylin and Eosin, black dotted circle = residual tumor cells. (**C**) Representative fluorescence images of tumor specimen in the operating room using intraoperative microscopes (Leica M720 and M530, Wetzlar, Germany) in white light, FL400 and FL800 modes. Scale bars, 2 mm (**D**) Representative fluorescence microscopy images of the brain tumor samples stained for Hsp70 (red), RAS70 peptide (green), DAPI (blue). (**E**) Comparison of visible fluorescence frequency of RAS70-Cy7.5 and 5-ALA in glioblastomas samples. Fisher test result **** *p* ≤ 0.0001, *ns* - not significant (**F**) Results of quantitative assessment of RAS70-Cy7.5, 5-ALA and NGL-RGD-Cy7.5 fluorescence in glioblastomas samples. Medians on the violin plot are indicated by the dashed horizontal line, Man–Whitney test result **** *p* ≤ 0.0001, VT = viable tumor zone, PZ = perifocal zone

In 27 viable tumor samples and 20 perifocal zone samples from glioblastoma, RAS70-Cy7.5 fluorescence was detected in 100% of cases, while 5-ALA fluorescence was visible in 23 (85.18%) and 6 (30%, *p* < 0.0001) samples, respectively (Fig. 3C, E). Quantitative analysis revealed significantly higher target-to-background ratio (TBR) fluorescence intensity with RAS70-Cy7.5 compared to 5-ALA in both viable and perifocal glioblastoma zones: 7.0 a.u. (4.8–13.2) vs. 1.8 a.u. (1.5–2.2) and 5.0 a.u. (4.8–9.6) vs. 0.3 a.u. (0.1–1.6), respectively (Fig. 3F). Viable tumor samples treated with RAS70 exhibited significantly higher visible fluorescence intensities compared to those treated with the control scramble peptide NGL-RGD-Cy7.5: 7.0 a.u. (4.8–13.2) vs. 0.9 a.u (0.72–1.9), *p* ≤ 0.0001 (Fig. 3F).

### Intraoperative fluorescence ex vivo imaging using peptide RAS70-Cy7.5, control scramble peptide NGL-RGD-Cy7.5 and 5-ALA on brain metastases samples

**Fig. 4:**
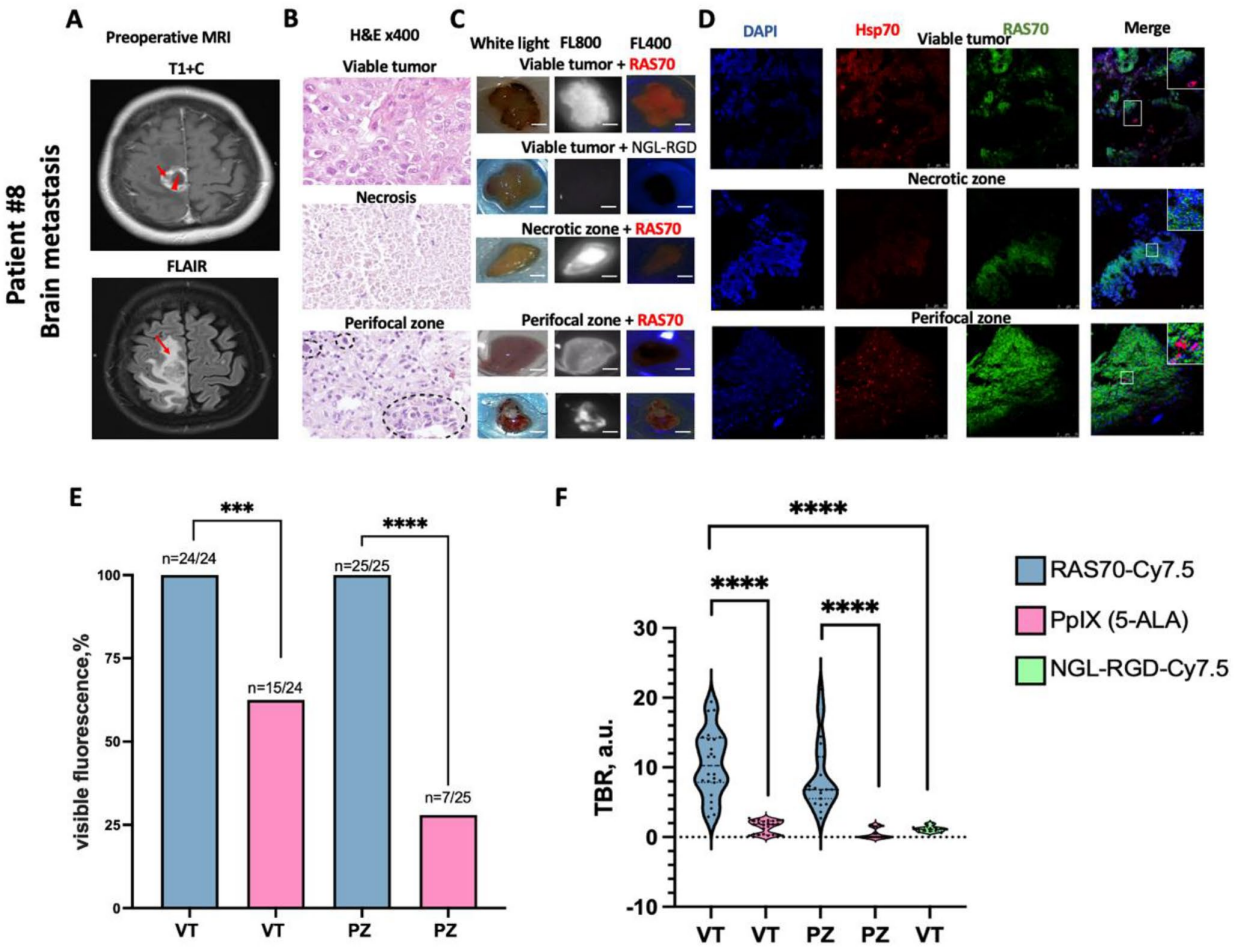
Representative intraoperative epifluorescence ex vivo imaging of brain metastasis (Patient #8). (**A**) Representative preoperative MR images show the sampling sites: on T1 + C image, the red arrow indicates the area of viable tumor tissue sampling, the red arrowhead indicates the area of necrosis, on the FLAIR image, the red arrowhead indicates the perifocal zone. (**B**) Representative histological images of viable tumor, necrosis, perifocal zone. H&E = Hematoxylin and Eosin, black dotted circle = residual tumor cells. (**C**) Representative fluorescence images of tumor specimen in the operating room using intraoperative microscopes (Leica M720 and M530, Wetzlar, Germany) in white light, FL400 and FL800 modes. Scale bars, 2 mm (**D**) Representative fluorescence microscopy images of the brain tumor samples stained for Hsp70 (red), RAS70 peptide (green), DAPI (blue). (**E**) Comparison of visible fluorescence frequency of RAS70-Cy7.5 and 5-ALA in metastases samples. Fisher test result *** *p* ≤ 0.001, **** *p* ≤ 0.0001. (**F**) Results of quantitative assessment of RAS70-Cy7.5, 5-ALA and NGL-RGD-Cy7.5 fluorescence in metastases samples. Medians on the violin plot are indicated by the dashed horizontal line, Man–Whitney test result **** *p* ≤ 0.0001, VT = viable tumor zone, PZ = perifocal zone

Of 24 viable tumor and 25 perifocal brain metastasis samples, visible fluorescence of RAS70-Cy7.5 was detected in 100% of cases, whereas 5-ALA fluorescence imaging showed visible fluorescence in 15 (62.5% *p* < 0.0001) and 7 (28%, *p* < 0.0001) samples, respectively (Fig. 4C, E). TBR index was statistically significantly higher when imaging with RAS70-Cy7.5 in both viable and perifocal zones compared with 5-ALA fluorescence: 10.25 a.u. (8–14) vs. 1.8 a.u. (0.4–2.3) and 6.8 a.u. (5.5–11.5) vs. 0.2 a.u. (0.1–1.7), respectively (Fig. 4F). As in the glioblastoma samples, the samples of viable zones from metastases treated with RAS70 showed a significantly higher intensity of visible fluorescence compared to the samples treated with the control scrambling peptide NGL-RGD-Cy7.5: 10.25 au (8–14) versus 1.1 au (0.85–1.35), *p* < 0.0001 (Fig. 4, F).

### Ex vivo fluorescence comparison of glioblastoma and metastasis samples

**Fig. 5:**
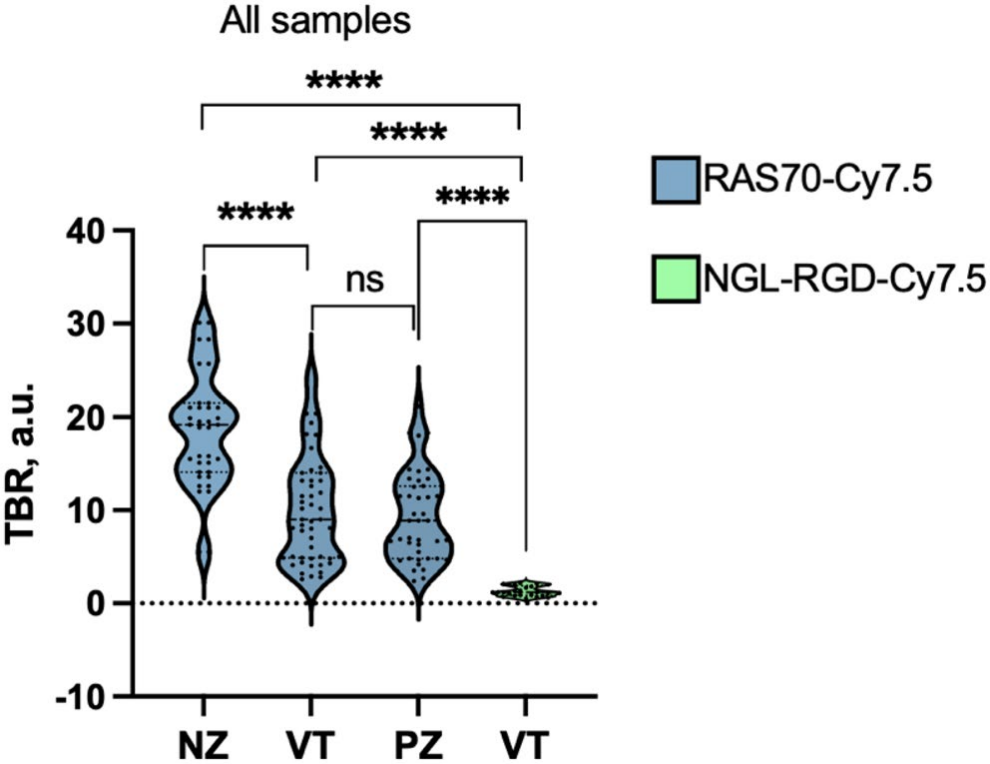
Results of quantitative assessment of RAS70-Cy7.5 and scramble peptide-Cy7.5 fluorescence in tumor tissue samples obtained from three zones. Medians on the violin plot are indicated by the dashed horizontal line, Man–Whitney test result **** *p*≤0.0001, ns - not significant; NZ= necrotic zone, VT = viable tumor, PZ = perifocal zone

The mean fluorescence intensities of RAS70-Cy7.5 did not significantly differ between high-grade glioma and metastasis specimens in either the viable tumor zones (7.0 a.u. (4.8–13.2) vs. 10.25 a.u. (8–14), *p* = 0.2) or the perifocal zone (5.0 a.u. (4.8–9.6) vs. 6.8 a.u. (5.5–11.5), *p* = 0.13). In the overall analysis of samples (*n* = 138) from two groups of tumors, the average TBR value in the neurotic zone was 19.2 a.u. (15.5–21.0), in the perifocal zone 8.9 a.u. (6.7–11.4), in the viable tumor 9.0 a.u. (7.0–11.5), which statistically significantly exceeded the TBR value in tumor samples (n-40) treated with the control scramble peptide 1.2 a.u. (0.9–1.7) (*p* < 0.0001; Fig. 5).

### Diagnostic accuracy of the Hsp70-based fluorescence imaging

**Fig. 6:**
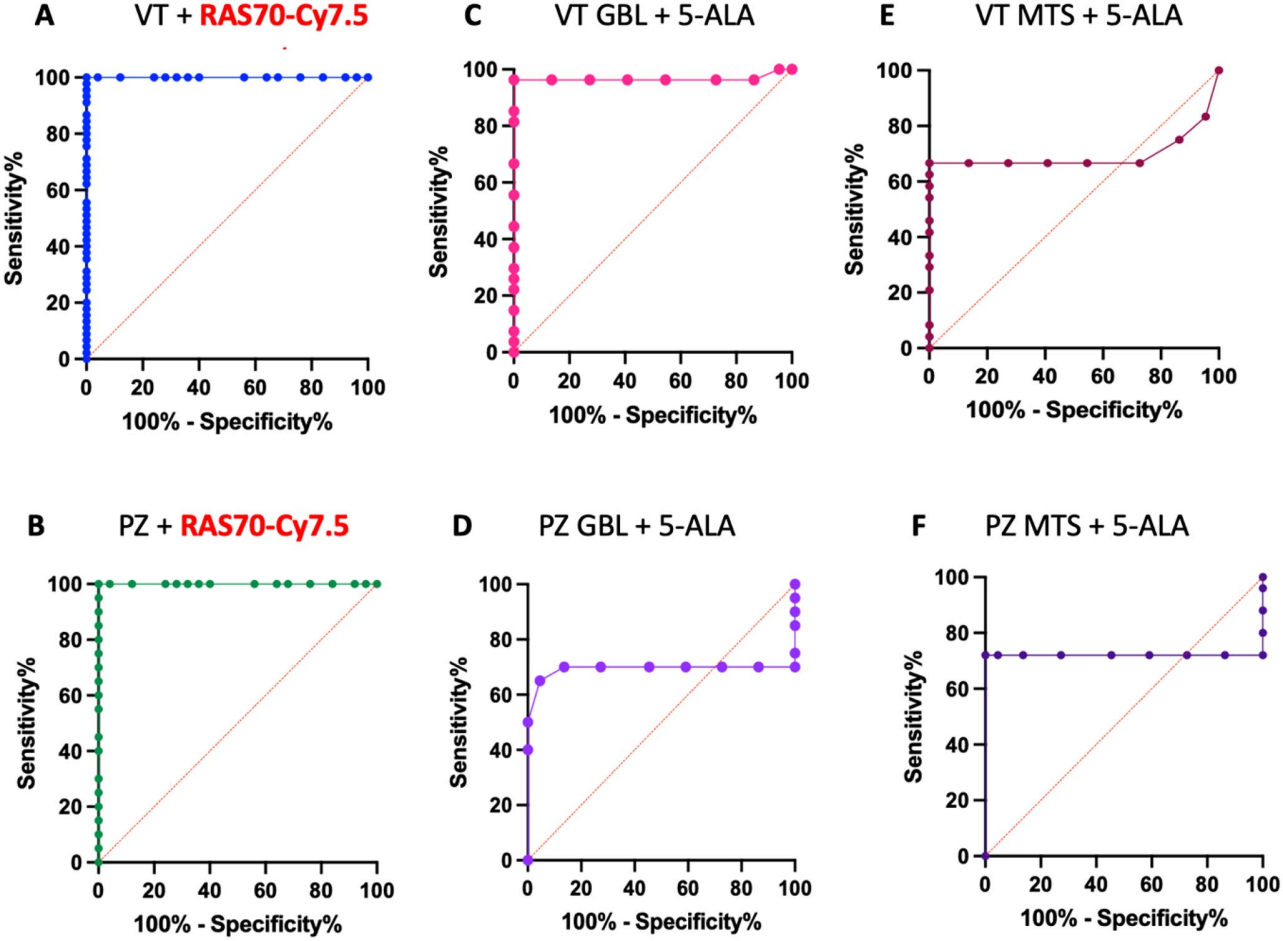
The diagnostic value of RAS70-C7.5 and 5-aminolevulenic acid in malignant brain tumors. VT = viable tumor, PZ = perifocal zone, GBL = glioblastoma, MTS = metastasis, 5-ALA = 5 -aminolevulinic acid

According to the ROC analysis, the optimal ratio of sensitivity and specificity (100% and 100% respectively) for detecting a viable tumor and perifocal tumor zone in fluorescence diagnostics using RAS70-Cy7.5 was observed at a TBR value of > 2.5 a.u. and > 2.3 a.u., respectively (Fig. 6A, B). The AUC values for the viable tumor and perifocal zone were 1.00 (95% CI, 1.00–1.00) and 1.00 (95% CI, 1.00 − 1.00) respectively, which characterizes the quality of the model as ideal.

5-aminolevulinic acid demonstrated high sensitivity and specificity (96.3% and 100% respectively) for fluorescence visualization of viable glioblastoma tissue. AUC value was 0.96 (95% CI, 0.90–1.00, optimal TBR threshold > 1.1.5 a.u. *p* < 0.0001), which characterizes the quality of the model as close to ideal (Fig. 6C). However, with regard to fluorescence diagnostics of the perifocal zone of glioblastoma, viable tissue and the perifocal zone of metastases, 5-ALA has a weak diagnostic value: AUC values of 0.69 (95% CI, 0.49–0.89, sensitivity 70.0% specificity 86.36%, optimal TBR threshold > 0.35 a.u. *p* = 0.03); AUC values of 0.69 (95% CI, 0.51–0.87, sensitivity 66.67%, specificity 72.73%, optimal TBR threshold > 0.85 a.u. *p* = 0.02); AUC values of 0.72 (95% CI, 0.54–0.89, sensitivity 72.00%, specificity 72.70%, optimal TBR threshold > 2.5 a.u. *p* = 0.009), respectively (Fig. 6D, E, F).

## Discussion

Over the past decade, intraoperative fluorescence imaging has emerged as a promising method for improving the accuracy of resection of malignant brain tumors [28, 29]. Currently, the FDA-approved 5-aminolevulinic acid drug for fluorescence imaging does not achieve high sensitivity and specificity for visual diagnostics of residual tumor cells in the perifocal zone of both malignant glial tumors and brain metastases, which requires the use of additional equipment for quantitative assessment of the degree of drug accumulation (e.g., biospectroscopy) [30]. In this regard, there is a need to develop agents for targeted fluorescence imaging that can be applied locally to tumor sites of interest without systemic administration into the body and that have high sensitivity and specificity for tumor cells, especially in the perifocal tumor zone. Herein, we demonstrated the diagnostic accuracy of such a targeted drug as the RAS70 peptide conjugated with infrared fluorophore Cy7.5 in relation to intraoperative ex vivo fluorescence imaging of malignant glial brain tumors and brain metastases.

Fluorescence intensity using the RAS70-Cy7.5 peptide, calculated using the TBR index, was statistically significantly higher in all tumor zones compared to using the control scramble peptide NGL-RGD-Cy7.5 (Figs. 3F, 4F and 5). The tumor-specificity of RAS70-Cy7.5 can be explained by the expression of the target molecule, mHsp70 on the surface of a high-grade gliomas and brain metastases as shown by live-cell immunofluorescence microscopy (Fig. 2). Notably, the distribution pattern of the RAS70 peptide corresponds to that of the TPP peptide, which other researchers have proposed for detecting mHsp70-positive breast and colorectal cancer tumors in mice [31–33]. Recent study by Holzmann et al. has shown that fluorescently labeled TPP used for the detection of mHsp70 also accumulated during local irrigation samples from human head and neck tumors [34]. However, its penetration depth into tumor tissue was only a 0.72 ± 0.03 mmIn our previous study, we observed a significantly greater depth of RAS70 penetration into tissues (3.0 ± 0.87 mm) compared to the Hsp70-targeting peptide without an RGD motif recently reported by Holzmann et al. [24]. We hypothesize that the presence of the RGD motif within the RAS70 tripeptide enhanced its tumor penetration capabilities.

Detailed analysis of peptide accumulation across various tumor zones revealed an approximately twofold higher concentration in the necrotic zone compared to both the contrast-enhancing and perifocal zones (Fig. 5). Indeed, during the destruction of the cytoplasmic membrane, heat shock proteins (including Hsp70) are released from the cytoplasm into the intercellular space, which subsequently leads to increased binding of the RAS70 peptide to the soluble chaperone [35]. It is worth considering that the peptide drug was applied to tumor samples outside the surgical wound, and the samples were cleared of blood and areas of coagulation necrosis caused by bipolar coagulation, since these factors in the surgical wound can potentially reduce the diagnostic accuracy of the RAS70. Bipolar coagulation used during surgery can presumably have a hyperthermic stress effect on tumor cells, which, as is known, can lead to an increase in the expression of membrane associated HSPs [36, 37]. However, the extent of this effect and its impact on the interpretation of data can only be determined during clinical and intraoperative studies. Now it can be noted that, as shown in previous studies, the time period from the moment of exposure to hyperthermia (> 42–43 °C) to the detectable increase in membrane-associated HSPs is more than several hours [38]. Therefore, it is likely that only in long-term, many hours surgeries should this factor be considered when analyzing images.

A clinically significant finding of this work is the detection of fluorescence RAS70-Cy7.5 in the perifocal tumor zone (≥ 3 mm from enhancing margin and within hyperintense FLAIR on preoperative MRI) that is comparable to the fluorescence intensity in viable tumor zones (Fig. 5). Histological examination revealed residual mHsp70-positive tumor cells in the perifocal zone of the tumor, which were not visually detectable by the surgeon during tumor removal. Using immunofluorescence microscopy, we noted the presence of mHsp70-positive tumor cells in the perifocal zone of both glioblastomas and metastases (Figs. 3D and 4D), and visual analysis of RAS70 peptide accumulation in the perifocal zone demonstrated effective tumor tissue contouring compared to the reference traditional fluorescence agent 5-ALA (Figs. 3C and 4C). The main reason why 5-ALA has a variable specificity is that the detection of fluorescence at the infiltrative margin is variable. According to ROC analysis, RAS70-Cy7.5 had higher sensitivity and specificity in diagnosing the perifocal tumor zone in both glioblastoma and metastases compared with 5-ALA (Fig. 6D, F).

It has been previously shown that the level of mHsp70 expression correlates with the invasive potential of a tumor by influencing cell migration, interacting with proteins involved in cytoskeletal remodeling, and binding to the extracellular matrix [22, 23, 39, 40]. Therefore, residual mHsp70-positive tumor cells in the perifocal zone may contribute to local and distant tumor recurrence. Also, we have previously demonstrated the colocalization of mHsp70 with markers of tumor stem-like cells (Nestin, SOX2) in glioblastoma cells [22, 41]. Stem tumor cells are known to provide not only glioblastoma relapse, but also resistance to conventional therapies [42, 43]. Targeting these cells with the RAS70 peptide, potentially in combination with a chemotherapeutic agent, may further prevent disease progression.

The targeted binding mechanism of the RAS70 peptide to tumor cells makes it an excellent complement to fluorescent agents with metabolic mechanisms of action (5-ALA) [7]. Furthermore, RAS70-Cy7.5 lacks the disadvantages associated with 5-ALA, such as the requirement for pre-operative systemic administration, precluding its use in emergency surgeries. The red fluorescence of protoporphyrin IX (emission 635 nm) shows greater absorption and scattering of light in tissues due to increased absorption by hemoglobin and water. Moreover, significant tissue autofluorescence in the visible spectrum, including the red region, can generate a high background signal and impede the detection of weak signals from fluorescent probes. Near-infrared fluorophores, such as RAS7-Cy7.5 (emission 800–900 nm), show significant background reduction and improvement in tumor-to-background ratio [44].

In our study, cohorts of studied subjects included patients with malignant glial tumors (*n* = 7) and with brain metastases (*n* = 8) of solid tumors of various histological types (including breast carcinoma, uterine carcinoma, colon adenocarcinoma, ovarian cancer, squamous cell lung cancer, lung adenocarcinoma). Сomparison of fluorescence intensity (TBR) indices using the RAS70 peptide on tumor biopsies from both groups revealed no statistically significant differences. Indeed, numerous previous studies clearly identified the presence of various membrane-bound HSPs, including Hsp70, on the surface of tumor cells [15, 17]. This makes RAS70-Cy7.5 a universal agent for fluorescence imaging of malignant tumors. It is also worth noting that our study included only patients with newly diagnosed brain tumors, but it is known that radiotherapy and chemotherapy can increase the expression of Hsp70 on the membrane of tumor cells [45]. In this regard, it can be assumed that the use of the RAS70 peptide in patients with recurrent brain tumors after complex treatment will be no less effective.

Currently, preclinical and clinical studies are investigating targeted fluorophores that bind to specific tumor epitopes, such as integrins or surface receptors (e.g., EGFR or VEGF). Near infrared fluorophores IRDye 800 CW (emits at 805 nm) conjugated to large molecules, such as antibodies to EGFR and VEGF receptors, have slower kinetics of incorporation into tumor cells, which requires taking the drug 1–5 days before surgery [46, 47]. Moreover, EGFR receptors are highly expressed only in 50–70% of GBL and therefore cannot be a universal diagnostic target [48]. Antibodies may also have immunogenic potential. Therefore, there is great interest in developing drugs based on fluorophores or nanoparticles that target tumor cells by adding small target peptides. Fluorophores conjugated to the integrin-targeting tripeptide RGD (arginine-glycine aspartic acid) (IRDye 800CW-RGD) or the protein tyrosine phosphatase mu-targeting peptide SBK2 (Cy5-SBK2) have shown a high fluorescence intensity ratio in glioblastoma cells compared to normal cells, but have not yet been demonstrated in vitro [49, 50]. In addition, molecular targeting peptides can be combined to form dual-targeting probes. In our study, the RAS70 peptide is a chimeric peptide consisting of the TKDNNLLGRFELSG peptide targeting mHsp70 and the RGD tripeptide, which promotes significantly higher uptake of the target peptide by tumor cells. While the control scramble peptide NGL-RGD showed 7-fold lower fluorescence intensity based on TBR value in viable tumor tissue.

### Limitations of the study

Our study has several limitations. First of all, this study was a prospective pilot study based on a small cohort of patients (7 patients with glioblastomas and 8 patients with brain metastases). However, despite the small number of patients, we were able to obtain 138 samples from three tumor zones for treatment with the RAS70 peptide, which allowed for statistical data processing. We also understand that the group of metastases was heterogeneous in the histological type of the primary tumor. But at the same time, we demonstrated the expression of mHsp70 and the staining of RAS70 samples obtained from metastases by fluorescence microscopy.

To exclude non-specificity of mHsp70 expression, we decided to select patients without brain tumors but who had undergone neurosurgical intervention as a control. We did not include patients with head trauma, since the presence of hemorrhages and contusion foci can lead to the release of Hsp70 into the intercellular space. Therefore, we decided to investigate mHsp70 expression in biopsy material after FCD resection in patients with drug-resistant epilepsy. Although only 2 patients with FCD were included, biopsy material was sufficient to conduct serial testing (*n* = 20).

In the article, we compare the fluorescence of RAS70-Cy7.5 with the fluorescence of 5-ALA (a precursor of protoporphyrin IX), that mainly validated for gliomas, not metastases. While 5-ALA and the RAS70 peptide have fundamentally different mechanisms of action, we chose 5-ALA as a comparator because it is widely used in clinical practice and FDA-approved. The targeted mechanism of action of the RAS70 peptide suggests that it could serve as an alternative or adjunct to 5-ALA, potentially improving imaging of the perifocal tumor zone. Although we conducted our experiment ex vivo, time from taking samples to experiment was around one minute. The samples could not have lost their viability in such a short period of time. First of all, we assessed the protoporphyrin IX fluorescence, and after sprayed samples of RAS70-Cy7.5 peptide. Furthermore, the RAS70-Cy7.5 peptide and protoporphyrin IX have distinct spectral properties (excitation/emission: 800 nm/820–900 nm and 400 nm/635 nm, respectively), thereby eliminating potential cross-talk between their fluorescence signals. While our research was not blinded, complete blinding is inherently challenging in studies involving intraoperative fluorescence imaging.

The main limitation of the ex vivo study is the lack of intraoperative conditions such as bleeding, the presence of cerebrospinal fluid, using of bipolar coagulation. In addition, topical use of the RAS70 peptide requires multiple reuses of peptides during surgery due to the small depth of penetration into tissue (3.0 ± 0.87 mm). Also, we used ImageJ software to analyse the fluorescence image contrast. However, this method is not applicable during surgery. We assume that the surgeon will evaluate the intensity of visible RAS70-Cy7.5 fluorescence visually or by biospectroscopy.

A prospective human clinical trial is planned to address these limitations and further evaluate the efficacy and safety of RAS70-Cy7.5 for intraoperative fluorescence imaging of malignant brain tumors.

In conclusion, it should be noted that RAS70-Cy7.5 is a fundamentally new method of intraoperative molecular diagnostics of tumor cells in viable tumor tissues. Compared with the reference fluorescence dye 5-ALA peptide, when using RAS70-Cy7.5, visible fluorescence of samples from the perifocal zone of glioblastomas and brain metastases is detected 3 times more often. At the same time, the sensitivity and specificity of RAS70-Cy7.5 by 30.0% and 13.64%, respectively, exceed the data of 5-ALA in diagnosing the perifocal zone in patients with glioblastoma when used, and by 28.0% and 27.30%, respectively, in diagnosing the perifocal zone in patients with metastases. RAS70-Cy7.5 also had 33.33% and 27.27% higher sensitivity and specificity, respectively, in detecting viable tumor tissue in patients with metastases compared to 5-ALA. The results of the ex vivo pilot study pave the way for further clinical trials in which the target RAS70 peptide will be applied topically to the surgical site. A prospective human clinical trial is planned to further evaluate the efficacy and safety of RAS70-Cy7.5 for intraoperative fluorescence imaging of malignant brain tumors.

## Data Availability

The datasets used and/or analyzed during the current study are available from the corresponding author Maxim Shevtsov on reasonable request.

## Abbreviations

ALA: Aminolevulinic acid
ATRX: Alpha thalassemia/mental retardation X–linked
DAPI: 4’,6–Diamidino–2–phenylindole
DWI: Diffusion–weighted imaging
EGFR: Epidermal growth factor receptor
FDA: Food and drug administration
FITC: Fluorescein isothiocyanate
FLAIR: Fluid–attenuated inversion recovery
Hsp: Heat shock protein
IDH: Isocitrate dehydrogenase
MGMT: O6–methylguanine–DNA methyltransferase
MRI: Magnetic resonance imaging
NZ: Necrotic zone
PBS: Phosphate–buffered saline
PpIX: Protoporphyrin IX
PZ: Perifocal zone
TMRM: Tetramethylrodamine methyl ester
TTF: Thyroid transcription factor
VT: Viable tumor
